# Consideration of Radioactive Iodine Therapy in a Graves' Disease Patient with Secondary Psychosis: A Case Report

**DOI:** 10.1055/s-0045-1813678

**Published:** 2025-12-18

**Authors:** Violerien U. Sultan, Budi Darmawan, Trias Nugrahadi, Achmad Hussein Sundawa Kartamihardja

**Affiliations:** 1 Department of Nuclear Medicine and Molecular Theranostics, School of Medicine, Universitas Padjadjaran/Dr. Hasan Sadikin General Hospital, Bandung, Indonesia

**Keywords:** Graves' disease, hyperthyroid, nuclear medicine, radioactive iodine, RAI, secondary psychosis

## Abstract

Psychosis is a rare but recognized neuropsychiatric complication of hyperthyroidism, most commonly associated with Graves' disease. Although anxiety and mood disturbances are more frequent, acute psychosis has been reported in approximately 1% of cases.

We report the case of a 30-year-old man with a 1-year history of Graves' disease who developed acute psychotic symptoms, including hallucinations and disorganized behavior. Laboratory findings revealed severe thyrotoxicosis with a suppressed thyroid-stimulating hormone and markedly elevated free T4. Thyroid scintigraphy using technetium-99m pertechnetate demonstrated diffuse increased uptake consistent with toxic diffuse goiter. The patient with severe thyrotoxicosis was initially managed with methimazole, propranolol, and psychiatric medications. Although the scheduled 1-week follow-up was missed, he returned at 4 weeks while continuing his prescribed therapy. At that visit, repeat thyroid function tests were obtained and psychiatric care was maintained. Subsequently, radioactive iodine (RAI) therapy at a fixed dose of 370 MBq (10 mCi) was administered after a short withdrawal of antithyroid drugs. One month posttreatment, both thyroid function and psychiatric symptoms improved significantly, allowing for discontinuation of antipsychotics. At 3 months, he became hypothyroid and was started on levothyroxine replacement.

This case highlights the importance of a multidisciplinary approach in managing patients with coexisting psychiatric conditions. Psychosis secondary to hyperthyroidism may mimic primary psychiatric disorders, potentially delaying diagnosis. Stabilizing psychiatric symptoms prior to definitive therapy and ensuring close psychiatric follow-up are essential to achieving optimal clinical outcomes. Radioiodine remains a safe and effective treatment for Graves' disease, even in patients with neuropsychiatric complications.

The psychotic symptoms in this case are caused by hyperthyroidism. RAI therapy can effectively and safely treat hyperthyroidism in Graves' disease patients with secondary psychosis.

## Introduction

Graves' disease is an autoimmune disease and is recognized as the most frequent etiology of hyperthyroidism. 1 Patients often present with a sudden onset of distinctive hyperthyroidism symptoms, including heat intolerance, weight loss, palpitations, irritability, and sleep disturbances. 2 3 The diagnosis of Graves' disease relies on clinical assessment, laboratory tests, and imaging studies. Laboratory evaluations typically include measurements of serum T3, T4, and thyroid autoantibodies. 4 While thyroid ultrasonography is generally sufficient for diagnosis, additional imaging techniques, such as radioactive iodine (RAI) uptake studies and neck computed tomography scans, may also be utilized. 2

Treatment for Graves' disease typically includes beta-blockers, antithyroid medications, and RAI. In cases where medical therapy proves ineffective, thyroidectomy may be necessary. 5 Graves' disease can also present with psychiatric symptoms, such as anxiety, depression, delirium, toxic psychosis, and bipolar disorders. Psychosis, though rare, occurs in approximately 1% of cases. 6 Proper diagnosis of such cases requires a thorough history and psychiatric evaluation to determine the severity of the disease. Treatment often involves antipsychotic medications alongside effective management of thyroid dysfunction, leading to significant improvement. 6

Graves' disease incident with psychiatric disorders in the form of psychosis is very rare in the Nuclear Medicine and Molecular Theranostics Department of Dr. Hasan Sadikin General Hospital, West Java, Indonesia. The following is a case of a 30-year-old male patient with Graves' disease accompanied by symptoms of psychosis. Administration of RAI therapy can overcome hyperthyroidism and symptoms of psychosis suffered by the patient. Based on the description above, the author would like to report the case of a 30-year-old man with Graves' disease who presented with secondary psychotic symptoms.

## Case Report

A 30-year-old male with a confirmed diagnosis of Graves' disease was referred to the Department of Nuclear Medicine at Dr. Hasan Sadikin General Hospital, Bandung, in February 2024. He had been on treatment with propylthiouracil (PTU) and propranolol for approximately 1 year. However, over the preceding month, he began exhibiting symptoms consistent with acute psychosis.

The patient reported smoking 12 to 14 cigarettes per day but denied any history of substance abuse, other major illnesses, or family history of hyperthyroidism or psychiatric disorders.

On physical examination, he appeared moderately cachectic, with a body mass index of 17.98 kg/m ^2^ . Notable findings included an enlarged neck, bilateral exophthalmos, excessive sweating, and fine tremors. Vital signs revealed a heart rate of 125 beats per minute, a respiratory rate of 20 breaths per minute, blood pressure of 120/70 mmHg, and a body temperature of 36.9°C. Psychiatric evaluation revealed paranoid ideation, both visual and auditory hallucinations, as well as disorganized behavior.

**Fig. 1:**
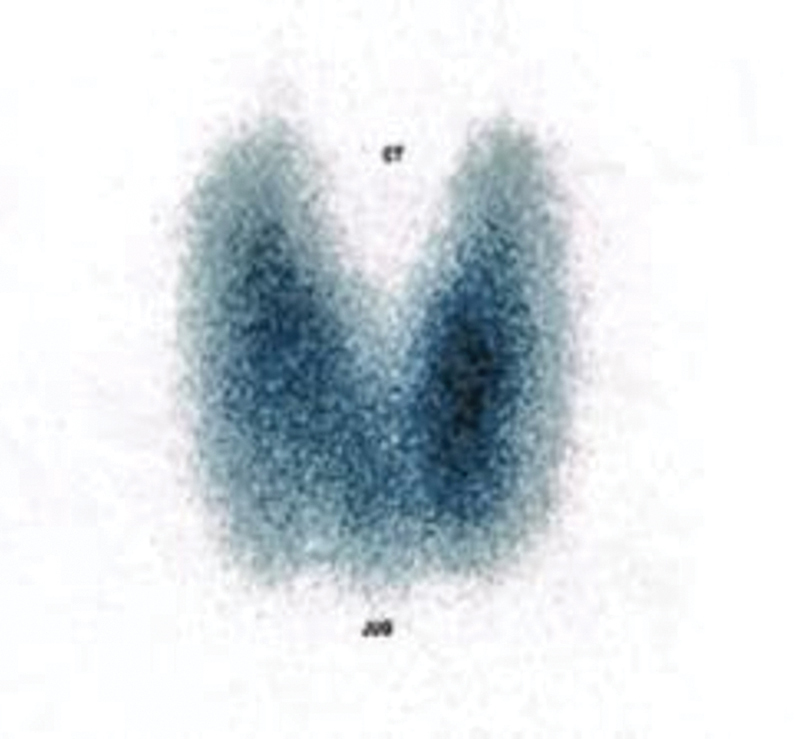
Thyroid scintigraphy using Tc-99m pertechnetate.

Laboratory investigations showed a suppressed thyroid-stimulating hormone (TSH) level of <0.003 µIU/mL (reference range: 0.350–4.940 µIU/mL) and an elevated free thyroxine (free T4) level of >5 ng/dL (reference range: 0.70–1.48 ng/dL). Thyroid scintigraphy using technetium-99m pertechnetate revealed diffuse increased uptake consistent with toxic diffuse goiter ( Fig. 1 ). Based on clinical, biochemical, and imaging findings, a diagnosis of Graves' disease-associated psychosis was established.

Given the severity of thyrotoxicosis, the patient was pretreated with methimazole (10 mg twice daily) and propranolol (10 mg twice daily) for 7 days. Concurrently, psychiatric management was initiated, consisting of haloperidol (5 mg twice daily), trihexyphenidyl (2 mg twice daily), and chlorpromazine (50 mg at night). The patient did not attend the scheduled follow-up visit at 1 week but eventually returned to the hospital 4 weeks later, during which he continued taking his prescribed medication. At that time, thyroid function tests (TSH and fT4) were repeated, and the patient was also referred for psychiatric follow-up. The results of TSH and fT4 are presented in Table 1 . RAI therapy was initiated at a fixed dose of 370 MBq (10 mCi) after a 3-day withdrawal of antithyroid medication, while continuing his psychiatric medications.

**Table 1:** Thyroid hormone levels and psychotic symptoms

Treatment		fT4 (0.7–1.8 ng/dL)	TSH (0.3–5.0 µIU/mL)	Psychotic symptoms
Before RAI	w/o pretreatment	>5 ng/dL	<0.003 µIU/mL	+
	With pretreatment	4.8 ng/dL	0.01 µIU/mL	+
After RAI	1 month	1.1 ng/dL	3.9 µIU/mL	−
	3 months	0.5 ng/dL	10.0 µIU/mL	−

Abbreviations: RAI, radioactive iodine; TSH, thyroid-stimulating hormone.

One month after RAI therapy, the patient's hyperthyroid symptoms showed marked improvement, and the psychiatric manifestations resolved completely. Psychiatric follow-up was maintained, which allowed for the gradual discontinuation of antipsychotic therapy. He was able to return to work and daily activities without further psychiatric complaints, as corroborated by his family. The clinical course and treatment response are summarized in Table 1 . At the 3-month follow-up, he developed hypothyroidism and was initiated on levothyroxine replacement therapy. Psychiatric follow-up continued for 6 months after RAI therapy; however, no further psychiatric medication was required.

## Discussion

Graves' disease, initially characterized by Robert Graves in 1835, is marked by a combination of thyroid enlargement, palpitations, ocular abnormalities, and neurological disturbances. 7 Although psychosis is a rare manifestation, it occurs in approximately 1% of cases. In the era prior to effective treatment options for hyperthyroidism, neuropsychiatric presentations such as psychosis and delirium were relatively common. 8 The advent of modern therapies has significantly improved disease control, leading to milder presentations, particularly in terms of psychiatric symptoms such as anxiety, depression, euphoria, and cognitive disturbances. 9

Current psychiatric classification systems recognize both hyperthyroid and hypothyroid states as contributors to mental health conditions, categorizing them as secondary to endocrine dysfunction. Notably, in some individuals, psychiatric symptoms may precede the clinical diagnosis of thyroid disease, with hyperthyroidism potentially acting as a trigger or aggravating factor. 9

The neuropsychiatric effects of hyperthyroidism are believed to result from elevated catecholaminergic activity due to increased density and sensitivity of beta-adrenergic receptors in both central and peripheral tissues. 10 11 12 This mechanism may account for the overlapping clinical features and the role of thyrotoxicosis in precipitating or intensifying psychiatric symptoms. Consequently, psychiatric symptoms are sometimes treated as primary conditions while hyperthyroidism is managed concurrently. Close interdisciplinary collaboration with psychiatrists is essential in such cases. 9

Given the possible association between Graves' disease and psychosis, thorough evaluation—including medical history, physical examination, psychiatric assessment, and laboratory testing—is critical to establish an accurate diagnosis and formulate an appropriate treatment plan. 9 A multidisciplinary approach helps determine the underlying etiology and optimizes patient outcomes.

Management strategies for hyperthyroidism typically fall into three categories 13 :

- Suppression of thyroid hormone production using antithyroid drugs (ATDs).
- Reduction or ablation of thyroid tissue via RAI therapy or surgery.
- Reducing the effects of thyroid hormones on peripheral tissues by administering beta-blockers.

Treatment selection depends on factors such as disease severity, patient age, goiter size, and coexisting medical conditions. While all three treatment modalities are effective for Graves' disease, patients with toxic adenoma or multinodular goiter typically respond better to RAI or surgical intervention due to the lower remission rates associated with ATDs. 13

In this case, the primary goal was to control the patient's thyrotoxicosis. After 1 year of ATD therapy with persistent hyperthyroidism and emerging psychotic symptoms, RAI was selected as definitive treatment. Methimazole was used as pretreatment due to its favorable safety profile, high efficacy, and long half-life, which permits once-daily dosing—making it ideal for reducing the risk of RAI-induced thyroid storm. 13

RAI therapy for Graves' disease typically involves a fixed dose ranging from 370 to 555 MBq (10–15 mCi), while toxic adenomas may require 10 to 20 mCi. Although calculated dosing based on thyroid gland volume and radioiodine uptake is available, it is more complex and less cost-effective. 13 14 Studies have shown no significant difference in clinical outcomes between fixed and calculated doses, with the former being more practical and yielding higher treatment success rates at 6 months. 14

Neuropsychiatric symptoms in hyperthyroidism generally improve following normalization of thyroid function. Initial management often involves a combination of ATDs and beta-blockers, which provide rapid symptomatic relief by mitigating adrenergic overactivity. 9 15 16 In cases where psychiatric symptoms persist or are severe, antipsychotics may be considered. 17 For example, Kathol et al observed that treating hyperthyroidism alone—regardless of the modality—was sufficient to resolve psychiatric symptoms in patients with comorbid major depression or anxiety disorder. 18 Similarly, Trzepacz et al reported complete psychiatric recovery in a cohort of women with newly diagnosed Graves' disease following antithyroid or RAI therapy combined with a beta-blocker. 19

It is also crucial to monitor for hypothyroidism following RAI, as this condition can itself cause psychiatric symptoms, including psychosis. Thyroid function testing is recommended 1 to 2 months post-therapy, and immediate initiation of levothyroxine is warranted upon confirmation of hypothyroidism. 20

## Conclusion

The psychotic symptoms in this case are caused by hyperthyroidism. RAI therapy can effectively and safely treat hyperthyroidism in Graves' disease patients with secondary psychosis.
